# Dendrites help mitigate the plasticity-stability dilemma

**DOI:** 10.1038/s41598-023-32410-0

**Published:** 2023-04-21

**Authors:** Katharina A. Wilmes, Claudia Clopath

**Affiliations:** 1 Imperial College London, London, United Kingdom; 2 Present Address: University of Bern, Bern, Switzerland

**Keywords:** Synaptic plasticity, Neuroscience, Computational neuroscience

## Abstract

With Hebbian learning ‘who fires together wires together’, well-known problems arise. Hebbian plasticity can cause unstable network dynamics and overwrite stored memories. Because the known homeostatic plasticity mechanisms tend to be too slow to combat unstable dynamics, it has been proposed that plasticity must be highly gated and synaptic strengths limited. While solving the issue of stability, gating and limiting plasticity does not solve the stability-plasticity dilemma. We propose that dendrites enable both stable network dynamics and considerable synaptic changes, as they allow the gating of plasticity in a compartment-specific manner. We investigate how gating plasticity influences network stability in plastic balanced spiking networks of neurons with dendrites. We compare how different ways to gate plasticity, namely via modulating excitability, learning rate, and inhibition increase stability. We investigate how dendritic versus perisomatic gating allows for different amounts of weight changes in stable networks. We suggest that the compartmentalisation of pyramidal cells enables dendritic synaptic changes while maintaining stability. We show that the coupling between dendrite and soma is critical for the plasticity-stability trade-off. Finally, we show that spatially restricted plasticity additionally improves stability.

## Introduction

Hebbian plasticity is considered to be the neural hallmark for learning and memory. It enables the formation of cell assemblies as it strengthens connections between cells with correlated activity. On the downside, correlations between cells are increased even further with Hebbian plasticity. Theoretically, such a positive feedback loop leads to undesired unstable runaway activity^1^. Cortical cells, however, fire at low rates in an asynchronous irregular manner. It is therefore unclear how neural activity in the functioning brain remains stable despite Hebbian plasticity. To resolve this dilemma, it has been suggested that homeostatic processes keep the network activity stable^2^. Homeostatic processes, such as homeostatic scaling^3–8^ or inhibitory plasticity^6,9–15^, counteract increases in the network activity, but it has been proposed that they might be insufficient to keep the network activity stable for the following reason: these processes operate on a timescale of hours or days^16–20^, but theoretical models require homeostatic mechanisms that act on the same timescale as Hebbian plasticity or faster^21–26^. Zenke et al.^22^ therefore, proposed that there must be a fast compensatory mechanism. Such a mechanism could modulate plasticity itself^27^. Models requiring fast mechanisms typically assume that plasticity is continuously happening^21,24^. In contrast, in the brain plasticity is highly regulated by different neuromodulators^28–34^, astrocytes^35^, and inhibitory interneurons^10,36,37^. These different regulators of plasticity can slow down, speed up, gate, or flip plasticity. They differ in their temporal and spatial precision and hence enable rigorous plasticity control. Another simple solution to counteract instability is limited synaptic strengths, or more elegantly a strong weight dependence^38^. While all these mechanisms are able to rescue stability, they come with the downside that they effectively limit the amount of plasticity. However, the theoretical studies investigating stability and plasticity in neural networks have neglected one important feature of neurons: their dendrites. Most excitatory synapses are located on the dendrites. Moreover, principal layer 5 pyramidal cells have large dendritic trees, which are electrotonically separate from the soma. Plasticity in these cells can be gated separately in the dendrite and the soma by separate inhibitory cell types^39,40^, or local neuromodulation. Furthermore, dendrites seem to become decoupled from somata during memory consolidation^41^. We, therefore, investigate whether gating of plasticity in dendrites can enable synaptic weight changes without strongly impairing the stability of network dynamics.

## Results

**Figure 1:**
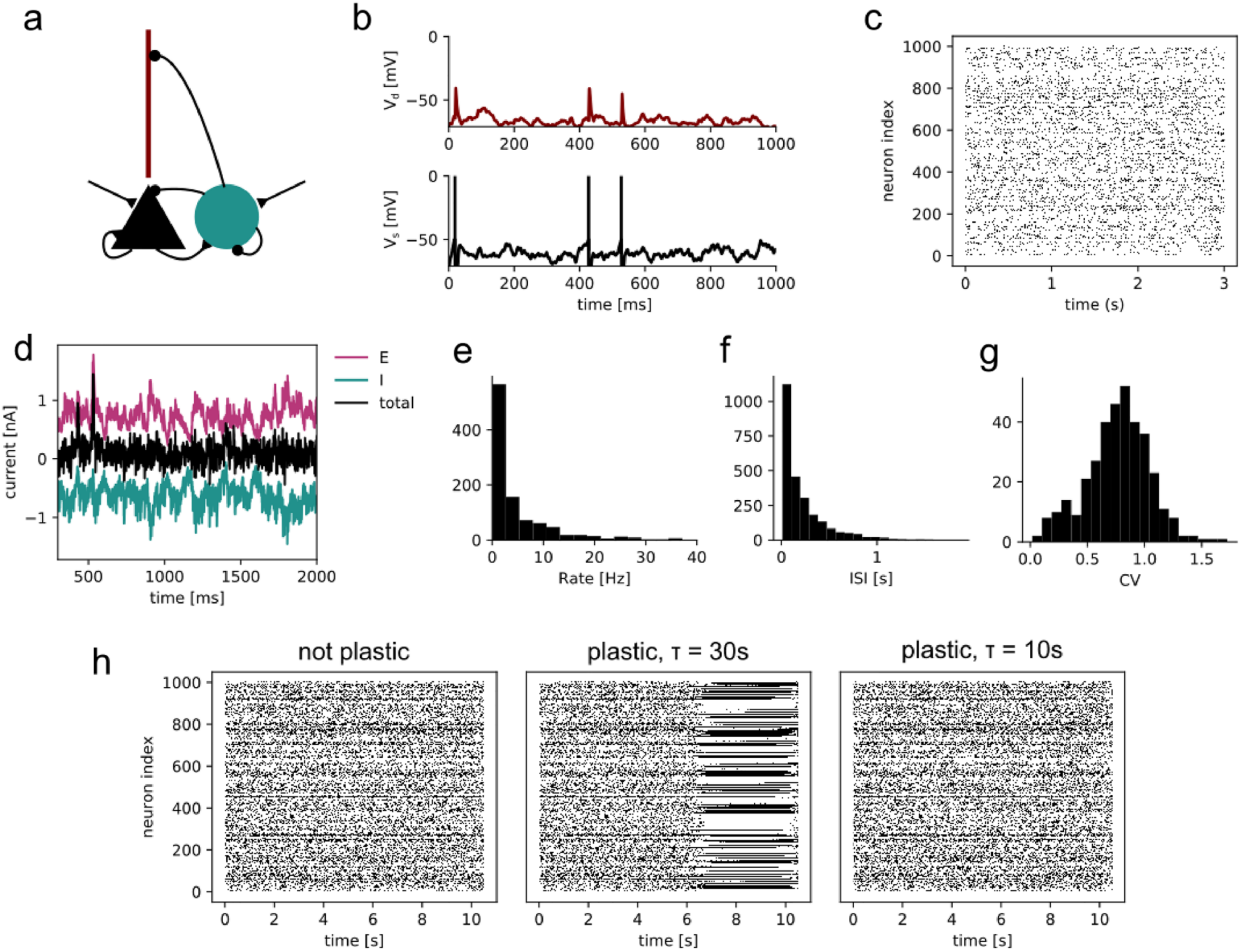
Balanced spiking neural network with 2-compartment pyramidal cells. (**a**) The network consisted of 1000 recurrently connected 2-compartment pyramidal cells (triangle and stick), and 250 recurrently connected inhibitory cells (circle). Both the excitatory and the inhibitory population receive external Poisson inputs (black arrows). (**b**) Somatic (black) and dendritic (red) voltage traces from one example pyramidal cell. (**c**) Raster plot of excitatory cell activity in the network. (**d**) Example currents from one example pyramidal cell. It receives large E (magenta) and I (cyan) currents which cancel on average (black). (**e**) Distribution of excitatory firing rates. (**f**) Distribution of excitatory interspike intervals. (**g**) Distribution of coefficient of variation (CV) of the interspike intervals. e-g indicate that the network is in a balanced state. (**h**) Raster plots of excitatory network activity in a network without plasticity (left), with plasticity and a homeostatic time constant $\tau =30\hbox {s}$, and with plasticity and a homeostatic time constant $\tau =10\hbox {s}$.

### Balanced spiking neural network with 2-compartment pyramidal cells.

To study how different modulators of plasticity affect stability and plasticity in dendrites and somata, we built a balanced recurrent network of 1000 excitatory pyramidal cells (E) and 250 inhibitory cells (I, Fig. 1). To investigate the benefit of dendrites, we modelled the pyramidal cells with two compartments^42^, one for the soma and one for the dendrite (Fig. 1a,b). The somatic compartment represents the perisomatic region, i.e. the soma and the proximal basal and apical dendrites, which contains the perisomatic synapses. The dendritic compartment represents the distal apical dendrites, which we will refer to as the dendrite, which contains the dendritic synapses. Both populations receive Poisson spike trains as external inputs. Before implementing plasticity in our model, we made sure that the network is in the asynchronous irregular regime (Fig. 1 c,e,f,g), due to a balance between excitation and inhibition. That is, strong excitatory recurrent inputs were balanced by strong inhibitory feedback (inhibition-stabilized regime^43^). On the single-cell level, this is reflected in large excitatory and inhibitory currents, which cancel each other on average (Fig. 1d).

To test the effect of plasticity in our network, we added a standard triplet STDP rule^44–46^ to the excitatory connections. As this form of plasticity is Hebbian, it can lead to an explosion of activity in recurrent networks^1,16^. To keep the activity of the network in the balanced state despite ongoing plasticity, we included homeostatic plasticity^1,16^. Following previous work^21,44,45,47^, the homeostatic process in our network monitored the postsynaptic firing rate and adjusted long-term depression (LTD) to keep the neurons at their target firing rate. The time constant $\tau$ of this homeostatic process is critical for stability as it determines how quickly the homeostatic process reacts to changes in firing rate. If $\tau$ is too large, the homeostatic plasticity cannot compensate for the correlation-based weight changes and the network activity explodes (Fig. 1h middle). When $\tau$ is sufficiently small, the homeostatic plasticity maintains stability (Fig. 1h right). The homeostatic time constant is a measure of stability in our model. The larger the homeostatic time constant, the more stable the network dynamics. To understand the contribution of dendrites to the plasticity-stability trade-off, we explored in our model how gating plasticity in dendrites affects homeostatic time constants.

**Figure 2:**
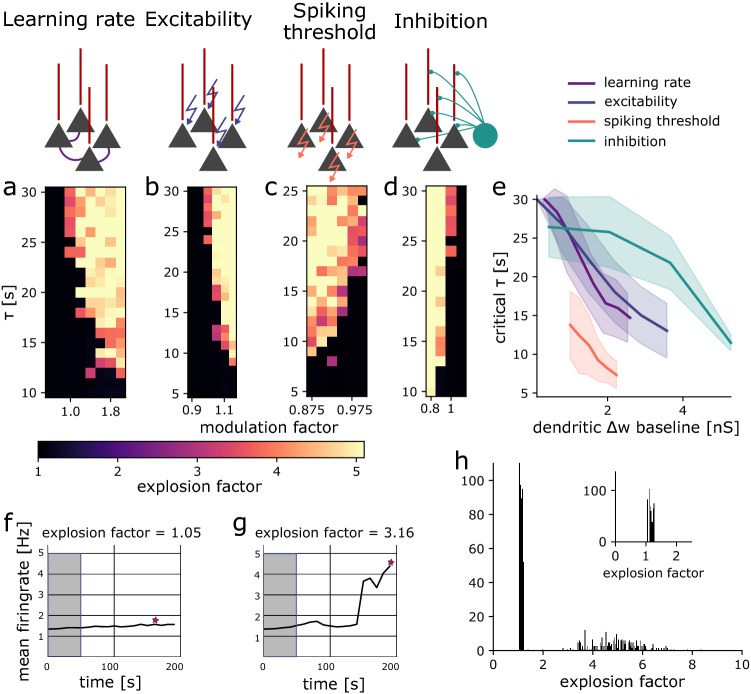
Gating plasticity increases stability. (**a**–**d**) Explosion factor as a function of homeostatic time constant $\tau$ and the respective gate (**a**) learning rate, (**b**) excitability, (**c**) spiking threshold (a measure of intrinsic excitability), (**d**) inhibition. (**e**) Comparison of the critical homeostatic time constant $\tau _{crit}$ for different gates, plotted as a function of baseline dendritic weight change to allow for comparison. (**f**–**g**) Illustration of the explosion factor. The star indicates the maximum firing rate of each simulation that was taken for the measurement of the explosion factor. The grey area denotes the reference firing rate at the beginning of the simulation, which was taken to calculate the explosion factor. (**f**) Example network simulation, where the firing rate does not explode (with explosion factor 1.05). (**g**) Example network simulation, where the firing rate explodes (with explosion factor 3.16). (**h**) Distribution of explosion factors. Inset: zoom into the x-axis.

### Gating plasticity increases stability

We next tested how different forms of plasticity modulation affect the required homeostatic time constant in our model. According to Hebbian plasticity, synapses change based on the pre- and postsynaptic activity, modulated by the learning rate. Therefore, plasticity can be modulated by changing the learning rate, or the firing rates of the connected cells. The firing rate depends on the excitatory and inhibitory currents to the cell and on the spiking threshold. Therefore, plasticity modulation via firing rate either targets those currents, or the threshold for spiking. In the following, we will list the different possibilities of plasticity modulation with reference to their biological counterpart.

The first gate we explored was the *excitability* of individual neurons to model the fact that neuromodulation can change the size or the duration of excitatory postsynaptic currents (EPSCs)^48^. In our two-compartment leaky-integrate-and-fire neuron model, we modelled excitability as a factor $\varvec{\gamma }$ multiplied to the excitatory synaptic currents (Eq. 1,2, the superscript *d* indicates the dendritic variable). The second gate was *inhibition*. We modelled its modulation by changing the inhibitory conductance $\varvec{g_I}$ (Eq. 1,2). The somatic and dendritic voltage ($V_s$ and $V_d$ respectively) was therefore modelled as

1 $$\begin{aligned} C_s \frac{dV_s}{dt}= & {} -g_l(V_s-E_l) - {\varvec{\gamma }} g_E V_s - {\varvec{g_I}} (V_s-E_I) + \lambda \left( g_s \frac{1}{1+\exp \left( -\frac{V_d-E_d}{D_d}\right) } + \omega ^{s}\right) \end{aligned}$$

2 $$\begin{aligned} C_d \frac{dV_d}{dt}= & {} -g_l^d(V_d-E_l) - {\varvec{\gamma ^d}} g_E^d V_d - {\varvec{g_I^d}} (V_d-E_I) + g_d \frac{1}{1+\exp \left( -\frac{V_d-E_d}{D_d}\right) } + c_d K(t) + \omega ^d \end{aligned}$$

where $C_{s/d}$ is the somatic/dendritic capacitance, $g_{I/E}$ is the inhibitory/excitatory conductance (index *d* indicates the dendritic variables), $E_l$/$E_I$ is the reversal potential of the leak/the inhibitory synapses (note that the reversal potential for the excitatory synapses is 0 mV and hence omitted), $g_s$ is the coupling from the dendrite to the soma, $\frac{1}{1+\exp (-\frac{V_d-E_d}{D_d})}$ is a nonlinear term for the dendritic calcium dynamics (see Methods), $\omega ^{s/d}$ is the somatic/dendritic adaptation variable, *K* the kernel for the back-propagating action potential current with amplitude $c_d$^42^. $\lambda$ ensures that the somato-dendritic coupling and adaptation are the same as in the model of^42^ (see Methods).

The third gate we explored was the *spiking threshold* $v_\theta$. When the somatic membrane potential $V_s$ reaches a threshold $v_\theta$, the neuron fires a spike. Spike times $t^f$ are therefore defined as $t^f : V_s(t^f)>v_\theta$. These three gates modulate plasticity indirectly by modulating the activity of the network. The fourth gate was *learning rate*, which modulates the synaptic weight changes directly, and was modelled as a factor $\varvec{\eta }$ in the weight update. Formally, the perisomatic synaptic weight $w_{ij}$ and dendritic synaptic weight $w^d_{ij}$ from neuron *j* to neuron *i* changed as:

3 $$\begin{aligned} \frac{d w_{ij}}{dt} =&{\varvec{\eta }} w_0 A^+ z_j^+ (t) z_i^{slow}(t-\varepsilon ) S_i(t) \nonumber \\&- {\varvec{\eta }} w_0 A_i^-(t) z_i^-(t)S_j(t) \end{aligned}$$

4 $$\begin{aligned} \frac{d w_{ij}^d}{dt} =&{\varvec{\eta ^d}} w_0 A^+ z_j^+ (t) z_i^{slow}(t-\varepsilon ) S_i(t) \nonumber \\&+ ({\varvec{\eta ^d}} w_0 ( -A_i^-(t) z_i^-(t) + A^{Ca} (v_d > \theta _{Ca})) - \alpha ) S_j(t) \end{aligned}$$

where $w_0$ is the initial weight, $A^{+/-}$ is the amount of potentiation/depression constants for the triplet rule, $\hbox {A}^{Ca}$ is the potentiation constant for the $\hbox {Ca}^{2+}$ spike-dependent potentiation, $S_{i/j}$ is the post-/presynaptic spike train, $z_j^+$ is the presynaptic trace, $z_i^-$ is the postsynaptic trace, $z_i^{slow}$ is the postsynaptic trace with slow time constant. $t-\varepsilon$ denotes that the value of the trace is taken before the action potential, which happened at time *t*. $v_d$ is the dendritic membrane potential. $\theta _{Ca}$ is the threshold for $\hbox {Ca}^{2+}$ spike-dependent plasticity (the term $v_d > \theta _{Ca}$ takes values 1 or 0 depending on whether $v_d$ is above the threshold $\theta _{Ca}$) and $\alpha$ is transmitter-induced depression.

To quantify how gating affects stability, we defined the explosion factor as the maximum firing rate in the simulation normalised by the firing rate at the beginning of the simulation, which indicates whether the network is stable (explosion factor close to 1, Fig. 2f) or explodes (explosion factor $>1.5$, Fig. 2g). The threshold of 1.5 for a network to be defined as exploding was based on the bimodal distribution of explosion factors (Fig. 2h).

We started by varying learning rate in both the perisomatic and the dendritic compartment (Fig. 2a). Expectedly, we found that with a low learning rate and a large homeostatic time constant $\tau$, the network was stable (the black region in Fig. 2a). For higher learning rates, the network activity exploded already at low values of $\tau$. This is expected as a higher learning rate increases the rate of synaptic change, which compromises the stability of the network. We defined the largest $\tau$ at which the network was still stable as the *critical homeostatic time constant*  $\tau _{crit}$ (Fig. 2a). A decrease in learning rate increased this critical time constant $\tau _{crit}$. Similarly, a decrease in excitability also increased the $\tau _{crit}$ (Fig. 2b). An increase in the spiking threshold has a similar effect as it makes the cells less likely to spike, i.e. less excitable (Fig. 2c). In these cases, $\tau _{crit}$ decreases with increasing excitability, as excitability increases the overall activity in the network, which in turn increases the amount of plasticity. An increase in inhibition on the contrary had the opposite effect on the critical time constant $\tau _{crit}$ (Fig. 2d). Increasing inhibitory inputs decreases firing rates in the network which improves network stability. In summary, homeostatic mechanisms for network stability can be slower when excitability and learning rate are downregulated or when inhibition is upregulated.

Although these effects were to be expected, qualitatively, our computational model allowed us to compare them quantitatively. We next characterised the different gates by comparing their effects on $\tau _{crit}$. To compare gates despite their different scales, we defined a common variable. That is, we plotted $\tau _{crit}$ as a function of the total dendritic weight change happening in a stable network (with a $\tau$ of 5 ms, see Methods, Fig. 2e). This analysis revealed that excitability and learning rate affect the critical time constant $\tau _{crit}$ in a different way than inhibition. $\tau _{crit}$ increases supralinearly as a function of the baseline dendritic weight change for the excitability and learning rate gates, whereas it increases sublinearly for the inhibition gate. Excitability and learning rate, hence, have a larger modulating gain than inhibition. For all gates, the actual dendritic weight change decreases as a function of the critical homeostatic time constant (Suppl. Fig. S1). Finally, by modulating different combinations of gating variables at the same time, we showed that they do not exhibit complex interaction effects (Suppl. Fig. S2). To conclude, all gates can improve network stability. However, they do so at the expense of synaptic weight changes.

**Figure 3:**
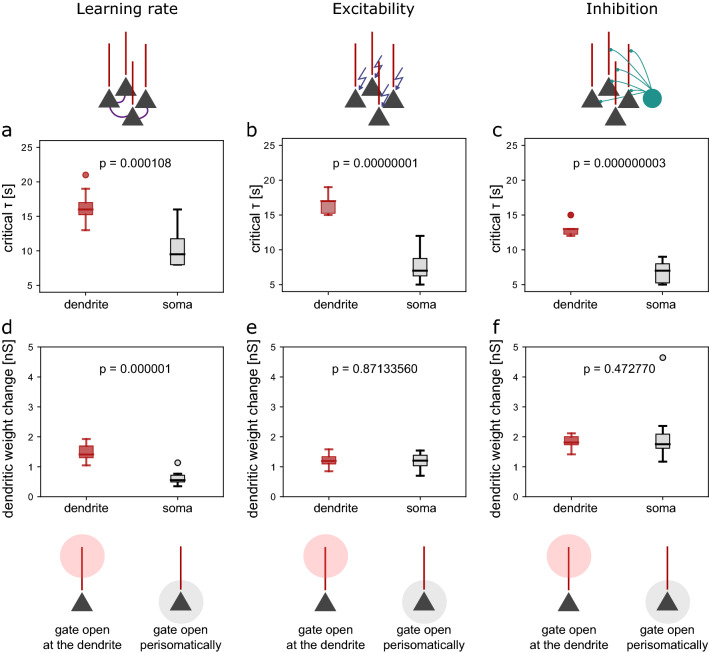
Learning in dendrites helps mitigate the plasticity-stability dilemma. (**a**–**c**) Distribution of critical homeostatic time constants for gating in the dendritic (red) and in the perisomatic (black) synapses for (**a**) a two-fold increase in the learning rate, (**b**) a 15% increase in excitability and (**c**) a 30% decrease in inhibition. (**d**–**f**) Distribution of dendritic weight changes for gating in the dendritic (red) and in the perisomatic (black) synapses for (**d**) a two-fold increase in the learning rate, (**e**) a 15% increase in excitability and (**f**) a 30% decrease in inhibition. The rectangles represent the interquartile range (IQR) between first and third quartiles. The thick horizontal lines represent the medians. The whiskers indicate the lowest and highest values within 1.5xIQR from the first and third quartiles, respectively. The circles denote outliers. All p-values were obtained by using the two-sample student’s t-test.

### Learning in dendrites helps mitigate the plasticity-stability dilemma

The increase in the critical time constant by gating comes at the cost of the lack of plasticity (measured as the total dendritic weight change, Suppl. Fig. S1). However, pyramidal neurons consist of a soma and a complex ramified structure of dendrites. Interestingly, the majority of excitatory synapses are located on dendrites, electrotonically distant from the soma. Inspired by these observations, we hypothesised that the anatomy of pyramidal cells could enable both plasticity of dendritic synapses and stable somatic activity at the same time. We, therefore, increased the learning rate and the excitability separately for the perisomatic and the dendritic synapses and compared their impact on $\tau _{crit}$.

We found that increasing plasticity (by increasing learning rate or excitability or decreasing inhibition) in the dendrite compromised the critical time constant $\tau _{crit}$ less than in the perisomatic compartment (Fig. 3). $\tau _{crit}$ was significantly larger for a two-fold increase in the learning rate in the dendrite than for the same increase in the learning rate in the perisomatic compartment (Fig. 3a). Moreover, modulating learning rate only in the dendrite allowed for significantly higher dendritic weight changes at a larger critical time constant (Fig. 3d). Increasing excitability by 15% in the dendrite led to a significantly larger $\tau _{crit}$ than increasing excitability by 15% in the perisomatic compartment (Fig. 3b), while there was no difference in dendritic plasticity between the two conditions (Fig. 3e). Similarly, a 30% decrease in dendritic inhibition maintained a significantly larger $\tau _{crit}$ than the same decrease in perisomatic inhibition (Fig. 3c), while there was no difference in dendritic plasticity (Fig. 3f). Note that we chose a two-fold increase in the learning rate, a 15% increase in excitability, and a 30% decrease in inhibition as these changes lower $\tau _{crit}$ by more than 50% (maximum explored values in Fig. 2e). Finally, we added inhibitory plasticity to the dendrite, which increases $\tau _{crit}$ even further (Suppl. Fig. S3a,c) without compromising dendritic weight changes (Suppl. Fig. S3b,d). The same amount of inhibitory plasticity added to a model without dendrites did not have such an effect (Suppl. Fig. S3).

In summary, by opening the gates for plasticity exclusively in the dendrite, the network can afford slower homeostatic mechanisms, higher network stability, while allowing the same or a higher amount of plasticity as when the gate is open at the perisomatic region.

**Figure 4:**
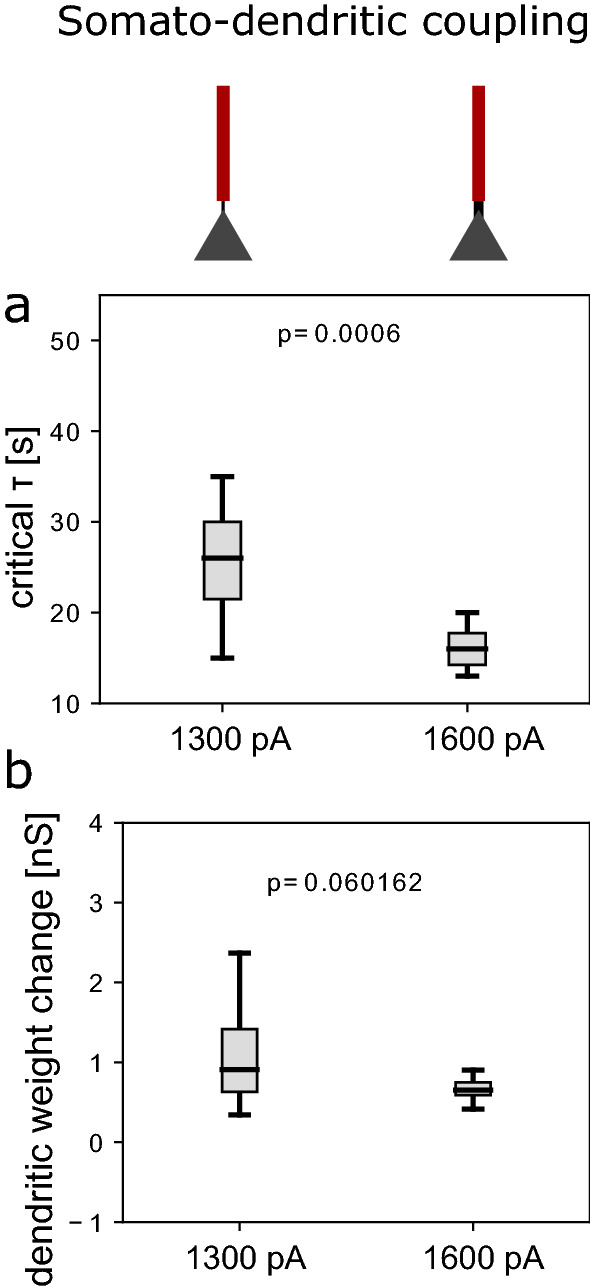
Effect of somato-dendritic coupling. (**a**, **b**) Effect of the dendrite-to-soma coupling, determined by $g_s$, i.e. how much the dendritic nonlinearity affects the soma. (**a**) Distribution of critical homeostatic time constants for a dendro-somatic coupling of 1300 pA (default) versus 1600 pA. (**b**) Distribution of dendritic weight changes for a dendro-somatic coupling of 1300 pA (default) versus 1600 pA. The rectangles represent the interquartile range (IQR) between first and third quartiles. The thick horizontal lines represent the medians. The whiskers indicate the lowest and highest values within 1.5xIQR from the first and third quartiles, respectively. The circles denote outliers. p-values were obtained by using the two-sample student’s t-test.

### Somato-dendritic coupling determines plasticity-stability trade-off

We hypothesised that the gain in stability resulting from gating plasticity in dendrites relies on the electrotonic separation of soma and dendrite. Interestingly, somato-dendritic coupling is dynamic and it has been shown that decoupling happens during REM sleep^41^. To test our hypothesis, we varied the coupling between soma and dendrite in our model. We found that an increase in coupling reduced the critical homeostatic time constant (Fig. 4), in line with our hypothesis. Another property that is special about the dendrite is the dendritic nonlinearity, which induces potentiation of dendritic synapses. Removing the nonlinearity from the model reduced dendritic synaptic weight changes and hence increased the stability of the network (Suppl. Fig. S4a,b). The combination of synaptic potentiation in the presence of a dendritic nonlinearity and the separation of soma and dendrite hence enables both dendritic weight changes and stable network dynamics. To further illustrate the effect of a dendritic compartment, we show that critical homeostatic time constants strongly decrease when opening the perisomatic gates in a network of single-compartment neurons (consisting of only a perisomatic compartment, Suppl. Fig. S5a,b). To conclude, the benefit of dendrites for synaptic plasticity while maintaining stability depends on the coupling between the dendrite and the soma.

**Figure 5:**
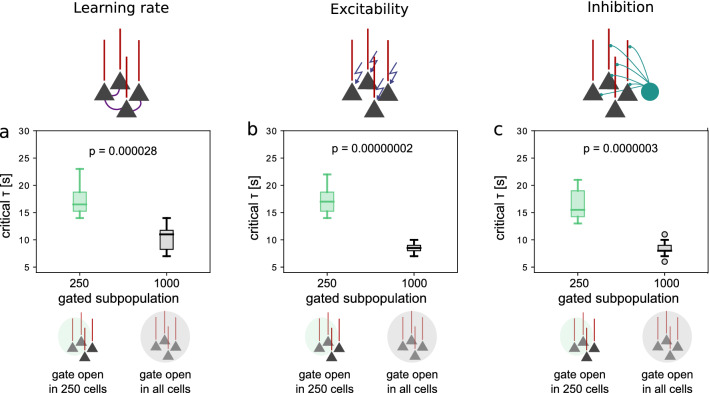
Spatially precise gating of plasticity enables learning while keeping network activity stable. (**a**) Distribution of critical homeostatic time constants for a two-fold increase in the learning rate in a subpopulation of excitatory cells (green) and in the entire network (black). (**b**) Distribution of critical homeostatic time constants for a 15% increase in excitability in a subpopulation of excitatory cells (green) and in the entire network (black). (**c**) Distribution of critical homeostatic time constants for a 20% decrease in inhibition in a subpopulation of excitatory cells (green) and in the entire network (black). The rectangles represent the interquartile range (IQR) between first and third quartiles. The thick horizontal lines represent the medians. The whiskers indicate the lowest and highest values within 1.5xIQR from the first and third quartiles, respectively. The circles denote outliers. p-values were obtained by using the two-sample student’s t-test.

### Spatially precise gating of plasticity enables learning while keeping network activity stable

Neuromodulators were typically thought of as global and diffuse^49^. However, neuromodulatory projections could in principle precisely target specific cell types and subpopulations, depending on their projective field and the receptor channels expressed in their targets. Specific neuromodulation^50^ could enable plasticity locally when learning requires synaptic adjustments only in a subset of neurons. To test how local gating of plasticity affects the critical time constant $\tau _{crit}$, we opened the gate for plasticity in only a subpopulation (one-fourth of the neurons) in the network and compared it to opening plasticity in the entire network.

We found that spatially confined plasticity had a much lower impact on the critical time constant than global plasticity. Here, we varied the gates in both the perisomatic and the dendritic compartment. An increase in the learning rate, an increase in excitability, or a decrease of inhibition lowered the critical time constant substantially (Fig. 5 a–c black). Opening these gates in only one-fourth of the neurons lowered the time constant significantly less than opening the gates in the entire network (Fig. 5 a–c green). Therefore, spatially confined gating of plasticity has advantages for network stability beyond enabling precise control.

## Discussion

We investigated the impact of a dendritic compartment on the plasticity-stability trade-off, by modeling different gating mechanisms in dendrites and somata in a spiking neural network and measuring their effect on stability and plasticity. Using a balanced spiking neural network with 2-compartment pyramidal cells, we showed how gating of plasticity increases stability. We found that excitability, learning rate, and inhibition affect the critical time constant in different ways. As hypothesised, the network was more tolerant towards weight changes, when plasticity gates were opened in the dendrite versus the perisomatic region. Plasticity in dendrites thereby could facilitate learning without compromising the stability of the network. We further showed that weak somato-dendritic coupling, as observed during REM sleep^41^, is required for the gain in stability. Finally, we showed that spatially precise gating of plasticity lifts the critical time constant and thereby could locally enable learning while keeping network activity stable.

It has been previously suggested that dendritic compartmentalisation together with dendritic nonlinearities serve network stability in the absence of plasticity^51^, increase signal-to-noise ratio^52^, and allow for various computations^53^. This is especially interesting as human pyramidal cells show enhanced compartmentalisation^54^. Here, we consider the role of dendrites for stability in the presence of plasticity.

Plasticity is highly modulated and gated^28–34,55,56^. In this paper, we explored different such modulations of plasticity. First, inhibitory cell types, which target perisomatic and apical dendrites of excitatory cells, can modulate plasticity. It has been shown that disinhibition - the inhibition of inhibitory cells - promotes learning^10,37,57^. Dendritic inhibition can influence plasticity directly by affecting depolarizing events in the dendrite, such as back-propagating action potentials and calcium spikes^40,58^. Perisomatic inhibition can modulate plasticity indirectly by decreasing the firing rate of the neuron, as synaptic weight changes depend on neural activity. The modulation of plasticity via inhibition can be both 1) fast, because interneurons can be switched on and off quickly, and 2) local, because they can be precisely targeted by fibers that provide cholinergic^59–63^ and noradrenergic neuromodulation^64,65^. Despite the importance of inhibitory cell types for diverse cellular and circuit computations, we did not find a significant difference for network stability when we included different forms of inhibition (feedforward versus feedback) to the perisomatic and the dendritic compartment (Suppl. Fig. S6). In addition, neuromodulators influence plasticity by regulating neural excitability (acetylcholine and noradrenaline^48,61,66^). For example, acetylcholine binds to muscarinic receptors, which activate a cascade that leads to a decreased permeability of potassium channels^48^. This prolongs the duration of EPSPs and thereby increases excitability. A similar form of neuromodulation is achieved by presynaptic inhibition. A recent theoretical study showed that presynaptic inhibition can act as a fast modulator of plasticity to stabilize network activity^27^. They showed that presynaptic inhibition is an attractive control mechanism as it depends on network activity and therefore provides a gain control loop. Similar to excitability in our model, the analysis in^27^ shows a supralinear relationship between presynaptic inhibition strength and the critical homeostatic time constant (Fig. 2). Finally, because many forms of plasticity are NMDAR-dependent^67^, a modulation of NMDA channels could affect plasticity directly. NMDA channel permeability can be modulated by D-serine, the origin of which is debated^68^, although it was initially thought to be synthesised by astrocytes^68,69^. Such a direct modulation of plasticity would correspond to modulation of learning rate in our model. With their slower kinetics^70^, NMDA channels could also contribute to stability. Therefore, we added NMDA channels to either excitatory, inhibitory, or both cell types, and found that the network is more stable if only excitatory cells contain NMDA channels (Suppl. Fig. S7a). With NMDA channels present in both populations or only in the inhibitory population, stability decreases (Suppl. Fig. S7b,c).

A different form of learning rate modulation could be achieved by dendritic inhibition, which is precisely timed to not affect the integration of EPSPs from the dendrite to the soma^40^. Localised gating could also be achieved by the interplay of multiple mechanisms or network effects. For example, non-specific gating together with the specific feedforward input could lead to specific activity-dependent gating (by a coincidence mechanism). Hence, plasticity is highly gated and modulated. Depending on the form of modulation, the effect on plasticity can be precisely timed and spatially confined.

The model makes the following experimentally testable predictions. We showed that larger synaptic changes are tolerated in dendrites than in the perisomatic region for the same critical time constant (Fig. 3). Therefore, our model predicts that more weight changes should be seen in dendrites. The weaker the dendrite and the soma are coupled, the larger becomes the advantage of the separate dendritic compartment. Hence, we predict that neurons with electrotonically more separate dendrites undergo more dendritic plasticity. Similarly, we predict that neurons with temporarily decoupled dendrites, such as pyramidal cells during REM sleep^41^ undergo more dendritic plasticity. Decoupled dendritic plasticity has also been shown during associative learning^39^. The decoupling could be achieved by dendritic inhibition^39,40,71^.

If plasticity is gated in space and time, i.e. synaptic changes are only locally permitted in limited periods of time, then we would observe that the total amount of synaptic change is not constant, but varies in time and space. The amount of synaptic change averaged over longer periods of time may be constant. When taking averages over shorter periods, we predict that the amount of synaptic change varies significantly over time.

Our model shows that the gates differ in their impact on the critical homeostatic time constant (Fig. 2e). We found that, for inhibition, the critical time constant decreases sublinearly as a function of the resulting increased dendritic weight change. For excitability and learning rate, however, the critical time constant decreases supralinearly. Our model, therefore, predicts that gating plasticity with inhibition allows for a larger critical time constant than gating plasticity with excitability or learning rate. We predict that when inhibition and excitability are separately modulated in an experiment, that the network will lose stability earlier with a change in excitability than with a change in inhibition.

We found that the gates also differ in their ability to protect memories (Suppl. Fig. S10). Learning rate is the only gate which can completely switch off plasticity to protect the memory. The memory breakdown increased supralinearly with a change in inhibition or excitability, whereas it increased linearly with a change in learning rate. Our model hence predicts that memories break down earlier when inhibition or excitability are modulated than with modulation of learning rate.

The homeostatic mechanisms which cause the dilemma reported by^22^ and our paper act on long time scales (hours to days) on the synaptic strengths, as e.g. the BCM sliding threshold and synaptic scaling. They ensure that synaptic weights do not grow unlimited. They can be considered homeostatic because they achieve a certain set point that is stable on average over long time scales. They are feedback controllers, which sense a recent average of the firing rate and adjust weights accordingly. To stabilise Hebbian plasticity, homeostatic mechanisms typically need to be as fast as or faster than the destabilising Hebbian plasticity^22^. Therefore, as^22^ point out, there must be other fast compensatory mechanisms in addition to those slow homeostatic mechanisms. Modelling studies used e.g. inhibitory plasticity with a fast timescale, or heterosynaptic or transmitter-induced plasticity to keep the models stable^24,72^. Inhibitory plasticity may have a stabilising role^73^, but the time scale of inhibitory plasticity appears to be rather slow in comparison to excitatory plasticity^62^. Presynaptic inhibition^27^ or intrinsic plasticity processes that act on the order of minutes^74,75^ are good candidates for fast compensatory mechanisms. For any such mechanism, it is however important that it does not destroy the signal or prevent plasticity altogether. To achieve both stability and plasticity, it is important that weight changes can occur. The homeostatic set point of weights should be achieved on average over longer time scales, while allowing temporal deviations from the setpoint^22^. The gates, we study here, especially excitability, spike threshold and inhibition could be the target of fast compensatory mechanisms. The point of our study, however, is that dendrites contribute substantially to the stability, which is often disregarded in modeling studies.

Our model provides a comparison between different gating mechanisms. The precise values for the critical homeostatic time constant depend on parameter choices (Suppl. Fig. S8). We simulated a balanced spiking network undergoing spontaneous activity to allow for the comparison of the different plasticity gates. A network which is externally stimulated has additional requirements for the homeostatic time constant, e.g. a network that receives plasticity-inducing stimuli (Suppl. Fig. S10).

In our model, we used one form of homeostatic plasticity, which adjusts LTD based on the postsynaptic firing rate. There are, however, different forms of homeostatic plasticity such as inhibitory plasticity^9^ and synaptic scaling^3^. Inhibitory plasticity also requires fast homeostatic mechanisms when plasticity is not gated^22,24^. With synaptic scaling as a homeostatic mechanism in our network (Suppl. Fig. S9), gating plasticity increases stability. We, therefore, expect that the gates studied here will similarly lift the requirements for the time scale of inhibitory plasticity and synaptic scaling. It will be interesting to explore the effects of the inhibitory gate on a homeostatic mechanism, which depends on inhibitory plasticity.

In summary, our study using balanced spiking neural networks with 2-compartment pyramidal cells shows how dendrites play an important role for the stability of neural networks in the presence of plasticity. Our results suggest an important role for a dynamic decoupling of dendrites from the soma as observed during learning^39^ and REM sleep^41^, which is important for memory consolidation^76^. Our results also imply that gating should be locally restricted, supporting the recent finding that neuromodulation may be more specific than initially thought^50^.

## Methods

### Balanced network

We built a recurrent neural network model with $N_E=1000$ excitatory (E) and $N_I=250$ inhibitory (I) cells. Both E and I cells received excitatory inputs from a pool of 1000 Poisson processes with a firing rate of 2 Hz and with a connection probability of p=10%. The E cells receive these inputs onto their perisomatic compartment. All neurons were randomly connected. Excitatory cells receive excitatory and inhibitory synapses on both their perisomatic and their dendritic compartment. The connection probability is 10% for all connections except from excitatory cells to excitatory cell’s perisomatic compartment. The connection probability for those connections is 9% to account for the fact that the cells also receive inputs on their dendrites in the two-compartment model. The connection strength of the synapses is chosen such that the network is balanced (see Table 1).

**Table 1:** Parameters of the network.

Parameter	Value
$N_E$	1000
$N_I$	250
$N_\text {Poisson}$	1000
$\lambda _\text {Poisson}$	2 Hz
*p*	0.1
$w_{EP}$	1.6 nS
$w_{IP}$	1.6 nS
$w_{EE}$	1.8 nS
$w^{d}_{EE}$	1.8 nS
$w_{IE}$	4.0 nS
$w_{II}$	6.0 nS
$w_{EI}$	8.0 nS
$w^{d}_{EI}$	4.0 nS

**Table 2:** Parameters of the neuron model.

Parameter	Value
$g_l$	10.0 nS
$g_l^d$	$\frac{170}{7}$ nS
$E_l$(leak)	− 70 mV
$E_I$(Inhibitory)	− 80 mV
$v_{theta}$	− 50 mV
$v_{theta}^I$	− 50 mV
$\tau _{m}$	20 ms
$\tau _{m}^I$	10 ms
$C_s$	200 pF
$C_d$	170 pF
$c_d$	2600 pA
$a_\omega ^d$	− 13 nS
$b_\omega ^s$	− 200 pA
$\tau _\omega ^s$	100 ms
$\tau _\omega ^d$	30 ms
$\tau ^s$	16 ms
$\tau ^d$	7 ms
$g_s$	1300 pA
$g_d$	1200 pA
$E_d$	− 38 mV
$D_d$	6 mV
$\lambda$	0.54

**Table 3:** Parameters of the plasticity.

Parameter	Value
$A^+$	6.5e − 3
$A^{Ca}$	7.2e − 2
$\alpha$	1e − 4
$\theta _{\text {bAP}}$	− 50 mV
$\theta _{Ca}$	− 40 mV
$\tau ^+$	16.8 ms
$\tau ^-$	33.7 ms
$\tau ^{slow}$	114 ms
$w_{\text {max}}$	10 nS
$\eta$	5

### 2-compartment pyramidal cell model

For the excitatory population, we used a 2-compartment integrate and fire pyramidal cell model with spike-triggered adaptation, adapted from the model by^42^ which was originally fitted to data from layer 5 pyramidal cells (see Table 2 for an overview of parameter values). It has two coupled membrane equations, one for the soma ($V_s$, Eq. 5), one for the dendrite ($V_d$, Eq. 6), modelled as (for clarity we repeat the equations from the main text):

5 $$\begin{aligned} C_s \frac{dV_s}{dt} = -g_l(V_s-E_l) - \gamma g_E V_s - g_I (V_s-E_I) + \lambda \left( g_s \frac{1}{1+\exp \left( -\frac{V_d-E_d}{D_d}\right) } + \omega ^{s}\right) \end{aligned}$$

6 $$\begin{aligned} C_d \frac{dV_d}{dt} = -g_l^d(V_d-E_l) - \gamma ^d g_E^d V_d - g_I^d (V_d-E_I) + g_d \frac{1}{1+\exp \left( -\frac{V_d-E_d}{D_d}\right) } + c_d K(t) + \omega ^d \end{aligned}$$

where $C_{s/d}$ is the somatic/dendritic capacitance, $g_{I/E}$ is the inhibitory/excitatory conductance (index *d* indicates the dendritic variables), $E_l$ and $E_l$ are the reversal potentials of the leak and the inhibitory synapses, respectively (note that the reversal potential of the excitatory synapses is 0 mV and, therefore, omitted), $\gamma$ is excitability, $\omega ^{d/s}$ is the somatic/dendritic adaptation variable. When the soma spikes, the dendrite receives a back-propagating action potential after a delay of 0.5 ms, which is modelled as a 2 ms long current pulse (defined by rectangular kernel *K*(*t*)) with amplitude $c_d=2600$ pA. With $\hat{t}_s$ as the time of the last somatic spike, *K*(*t*) is defined as

$$\begin{aligned} K(t) = \Bigl \{\begin{array}{ll} 1 &{} \text {if } \hat{t}_s + 0.5\text { ms} \le t \le \hat{t}_s + 2.5\text { ms}\\ 0 &{} \text {otherwise}.\\ \end{array} \end{aligned}$$

The dendrite has a nonlinear (sigmoidal) term $\frac{1}{1+\exp (-\frac{V_d-E_d}{D_d})}$ corresponding to the activation of dendritic calcium channels. $E_d$ determines the voltage at which the threshold will be reached and $D_d$ determines the slope of the nonlinear function. The nonlinear dynamics are controlled locally by $g_d$ and are also transmitted to the soma with a coupling factor $g_s$, such that the soma bursts. The factor $\lambda$ ensures that the somato-dendritic coupling and adaptation are the same as in the model of^42^, where the somatic capacitance was 370 pF (we used $C_s$=200 pF). The somatic adaptation variable is modelled as

7 $$\begin{aligned} \frac{d\omega ^{s}}{dt} = -\omega ^{s}/\tau _\omega ^s + b_\omega ^s S_i (t) \end{aligned}$$

where $b_\omega ^s$ is the strength of spike-triggered adaptation and $\tau _\omega ^s$ is the recovery time scale. The dendritic adaptation variable is written as

8 $$\begin{aligned} \tau _\omega ^d \frac{d\omega ^d}{dt} = -\omega ^d + a_\omega ^d (V_d - E_l) \end{aligned}$$

where $a_\omega ^d$ is the strength of subthreshold adaptation and $\tau _\omega ^d$ is the recovery time scale.

For the inhibitory population, we used a single-compartment leaky integrate-and-fire neuron model, which membrane potential *V* evolves according to:

9 $$\begin{aligned} C_s \frac{dV}{dt} = -g_l(V-E_l) - g_E V - g_I (V-E_I) \end{aligned}$$

For all neurons, excitatory and inhibitory conductances, $g_E$ and $g_I$ respectively, are increased by the synaptic weight $w_{iE}$/$w_{iI}$, depending on their type *i* upon a spike event in a presynaptic excitatory or inhibitory neuron with spike train $S_j(t)$, and decay exponentially with time constants $\tau _E$ and $\tau _I$, respectively:

10 $$\begin{aligned} \frac{dg_E}{dt}= & {} -\frac{g_E}{\tau _E} \end{aligned}$$

11 $$\begin{aligned} \frac{dg_I}{dt}= & {} -\frac{g_I}{\tau _I} \end{aligned}$$

Both excitatory and inhibitory neurons had a refractory period of 8.3 ms (chosen according to the network model from^21^). Initial membrane potentials for $V_s$ and *V* were sampled from a Gaussian distribution with $\mu$ = −70 mV and $\sigma$ = 10 mV to prevent that all neurons spike at the same time at the beginning of a simulation. $V_d$ was set to −70 mV initially.

### Plasticity

Synapses from neuron *j* targeting the perisomatic compartment of neuron *i* change their synaptic weight $w_{ij}$ according to the triplet rule^44^ (see Table 3 for an overview of parameter values). For clarity we repeat the same equation as in the main text:

12 $$\begin{aligned} \frac{d w_{ij}}{dt} =&\eta w_0 A^+ z_j^+ (t) z_i^{slow}(t-\varepsilon ) S_i(t) \\&- \eta w_0 A_i^-(t) z_i^-(t)S_j(t) \end{aligned}$$

where $w_0$ is the initial weight, $A^{+/-}$ is the amplitude of potentiation/depression (the depression one is time dependent, see below), $S_{i/j}$ is the post-/presynaptic spike train, $z_j^+$ is the presynaptic trace, $z_i^-$ is the postsynaptic trace, $z_i^{slow}$ is the postsynaptic trace with a slower time constant. $\varepsilon$ denotes a small fraction of time such that $t-\varepsilon$ indicates that the value of the trace is taken before the time point of the action potential *t*. Parameters were chosen as in^21^.

Synapses from neuron *j* to neuron *i* targeting the dendritic compartment change their synaptic weight $w_{ij}^d$ according to the same triplet rule with the back-propagating action potential (bAP) as the postsynaptic spike and an additional Ca-spike-dependent potentiation at the time of a presynaptic spike.

13 $$\begin{aligned} \frac{d w_{ij}^d}{dt} =&\eta ^d w_0 A^+ z_j^+ (t) z_i^{slow}(t-\varepsilon ) S_i^{\text {bAP}}(t) \\&+ (\eta ^d w_0 ( -A_i^-(t) z_i^-(t) + A^{Ca} (v_d > \theta _{Ca})) - \alpha ) S_j(t) \end{aligned}$$

Here, the timing of the back-propagating action potential in the dendrite is used to update the post-synaptic traces $z_i^-$ and $z_i^{slow}$ and $S_i^{\text {bAP}}(t)$ is the postsynaptic train of back-propagating action potentials. A back-propagating action potential is detected if three conditions are met: (1) the dendritic membrane potential $v_d$ exceeds a threshold of -50 mV, and (2) there was a somatic spike within the last 3 ms, and (3) there was no backpropagating action potential within the last 5.8 ms (to account for the refractory period). Synapses are potentiated by a constant amount $A^{Ca}$ when the presynaptic cell fires and the postsynaptic dendritic membrane potential $v_d$ exceeds a threshold $\theta _{Ca}$ of − 40 mV. The term $v_d > \theta _{Ca}$ takes a value of 1 when the threshold is crossed and is 0 otherwise. Synapses are depressed by a constant amount $\alpha$ for each presynaptic spike (transmitter-induced plasticity).

The pre- and postsynaptic traces are defined as:

14 $$\begin{aligned}&\frac{dz_j^+}{dt} = -\frac{z_j^+}{\tau ^+} + S_j(t) \end{aligned}$$

15 $$\begin{aligned}&\frac{dz_i^-}{dt} = -\frac{z_i^-}{\tau ^-} + S_i(t) \end{aligned}$$

16 $$\begin{aligned}&\frac{dz_i^{\text {slow}}}{dt} = -\frac{z_i^{\text {slow}}}{\tau ^{\text {slow}}} + S_i(t) \end{aligned}$$

where $\tau ^+$, $\tau ^-$, and $\tau ^{\text {slow}}$ are the time constants with which the traces decay. Both perisomatic and dendritic excitatory synapses are limited by a maximum synaptic weight $w_{\text {max}}$ = 10 nS.

#### Homeostatic plasticity

The depression amplitude $A^{-}_i$ for all synapses onto neuron *i* is a function of a moving average of neuron *i*’s activity $\bar{s}_i$:

17 $$\begin{aligned} A^{-}_i(t) = \frac{A^+ \tau ^+ \tau ^{\text {slow}}}{\tau ^- \kappa } \bar{s}_i^2 \end{aligned}$$

where $\kappa$ is the target firing rate, $A^+$, $\tau ^+$, $\tau ^-$ and $\tau ^{\text {slow}}$ are variables from the triplet STDP rule and $\bar{s}_i$ is the low-pass filtered spike train:

18 $$\begin{aligned} \tau \frac{d\bar{s}_i}{dt} = - \bar{s}_i + S_i(t) \end{aligned}$$

with $\tau$ defining the time constant of the homeostatic plasticity.

### Explosion factor

We quantify the stability with the explosion factor EF. We calculate it as follows:

19 $$\begin{aligned} EF = \frac{r_{max}}{r_{baseline}} \end{aligned}$$

where $r_{max}$ is the maximum population firing rate within the duration of the simulation and $r_{baseline}$ is the population firing rate averaged over the first 50 s of the simulation. Therefore, an explosion factor close to 1 indicates that the network activity is stable. The distribution of explosion factors was bimodal with a sharp peak close to 1 and a broader distribution of larger EFs (Fig. 2h). We defined a threshold separating those two modes, which defines whether the network is stable or explodes:

$$\begin{aligned} \text {network is }\Bigl \{\begin{array}{lr} \text {stable} &{} \text {if } EF \le 1.5\\ \text {unstable} &{} \text {if } EF > 1.5.\\ \end{array} \end{aligned}$$

### Critical time constant $\tau _{crit}$

For each value of the gate, we calculated the maximum $\tau$ for which the network was stable. It additionally had to be smaller than the minimum $\tau$ for which the network was unstable.

### Baseline dendritic weight change

For each gating value, we calculated the sum of all weight changes in the dendrite in a 200 s simulation with a $\tau$ of 5 s.

### Statistical analyses

To test for significance in Figs. 3 and 5, we used the two-sample two-sided student’s t-test.

### Simulation

All simulations were done with the Brian 2 simulator^77^. For Fig. 1, we simulated the network for 10 s without plasticity. Simulations to calculate the explosion factor (for Figs. 2, 3, 4, 5) were run for 200 s. We simulated an initial warm-up phase for 3 $\tau$ seconds without plasticity to calculate the average population firing rate for the balanced network. We used the average population firing rate of the last 2 s of the warm-up phase to set the target firing rate $\kappa$ in our model. We then switch on plasticity. All simulations were run at a timestep of 0.1 ms. For the plots in Figs. 1-3, each condition was simulated with 10 different seeds.

## Supplementary Information

Supplementary Information.

## Data Availability

The simulation code is available on GitHub at https://github.com/k47h4/Dendrites.
