# Supplementary material for: Dendrites help mitigate the plasticity-stability dilemma
Source: Sci Rep. 2023 Apr 21;13:6543. doi: 10.1038/s41598-023-32410-0 (PMC10121616; doi:10.1038/s41598-023-32410-0)
Supplement: Supplementary file 1 — Supplementary Information. [file 41598_2023_32410_MOESM1_ESM.pdf]

Supplementary information
Dendrites help mitigate the plasticity-stability dilemma

Katharina A. Wilmes[1,†], Claudia Clopath[1]

1 Bioengineering Department, Imperial College London, SW72AZ, London, UK
† present address: Department of Physiology, Universität Bern, Bühlplatz 5, 3012 Bern, Switzerland

Supplementary Methods

Inhibitory Plasticity

Synapses from inhibitory neuron j to excitatory neuron i change their weight w_{ij}^I according to Vogels *et al.* (2011)

$$\begin{aligned}\frac{dw_{ij}^I}{dt} =&\eta^I z_j^I(t) S_i(t) \\ &+ (\eta^I z_i^I(t) - \alpha^I) S_j(t)\end{aligned} \tag{1}$$

where η^I is the inhibitory learning rate, $z_j^I(t)/z_i^I(t)$ is the pre/post-synaptic trace, $S_j(t)/S_i(t)$ is the pre/post-synaptic spike train, and $\alpha^I = 2\kappa\tau_{iSTDP}$ determines the amount of transmitter-induced depression (Vogels *et al.* 2013). κ is the target firing rate, which was determined by the average population firing rate of the last 2 s of the warm-up phase. Plastic inhibitory weights are limited by a maximum weight $w_{\text{max}}^I = 100\text{nS}$. The pre/post-synaptic traces $z_j^I(t)/z_i^I(t)$ are written as

$$\frac{dz_j^I}{dt} = -\frac{z_j^I}{\tau_{\text{iSTDP}}} + S_j(t) \tag{2}$$

$$\frac{dz_i^I}{dt} = -\frac{z_i^I}{\tau_{\text{iSTDP}}} + S_i(t). \tag{3}$$

Table 1: **Parameters of the inhibitory plasticity.**

Parameter	Value
τ_{iSTDP}	20 ms
w_{max}^I	100 nS
η^I	0.05
α^I	$2\kappa\tau_{iSTDP}$

Inhibitory plasticity in networks with dendrites and networks without dendrites

For Suppl. Fig. S3, all parameters were the same as in the original network (see Methods and Parameter Tables in the main manuscript), if not mentioned otherwise in the following: For the network without dendrites, we used the same parameters, except that g_s was set to 0 and we placed the excitatory and inhibitory synapses that used to be on the dendrite in the original network (w_{EE}^d and w_{EI}^d) onto the soma. For the simulations with inhibitory plasticity, we added inhibitory plasticity (see section "Inhibitory Plasticity") only to those synapses that are on the dendrite (w_{EI}^d) in the model with dendrites, or to the fraction of additional synapses (that used to be on the dendrite) on the soma in the model without dendrites. In all simulations, the learning rate η of excitatory plasticity was doubled to 10.

Gating plasticity in two-compartment versus single-compartment networks

For Suppl. Fig. S5, we simulated a network of the same size, where excitatory cells were single-compartment neurons. The somatic membrane equation was the same as for the two-compartment neurons with $g_s = 0$ (no coupling to the dendrite). All parameters were the same as in the original network, with two exceptions, as we placed all synapses on the perisomatic compartment. First, the connection probability from excitatory to excitatory cells was increased to $p_{EE} = 0.19$ to account for the additional synapses that were previously placed on the dendrite. Second, in addition to the inhibitory synapses the soma already contained in the original simulation, it contained inhibitory synapses with the connection strength of inhibitory synapses on the dendrite in the original model $w_{EI}^d = 4.0nS$ with a probability of 0.1.

Network with inhibitory subtypes

For Suppl. Fig. S6, we included two populations of inhibitory celltypes, corresponding to parvalbumin-positive (PV) and somatostatin-positive (SST) interneurons. The PVs exclusively target the soma of the excitatory cells, and the SSTs exclusively target the dendrites of the excitatory cells. Excitatory cells and PVs, but not SSTs received feedforward Poisson inputs.

Parameter	Value
N_E	1000
N_{SST}	250
N_{PV}	250
N_{Poisson}	1000
λ_{Poisson}	2 Hz
p	0.1
$w_{E,Poisson}$	1.6 nS
$w_{PV,Poisson}$	1.6 nS
w_{EE}	1.8 nS
w^d_{EE}	1.8 nS
$w_{PV,E}$	4.0 nS
$w_{SST,E}$	4.0 nS
$w_{PV,PV}$	6.0 nS
$w_{SST,SST}$	6.0 nS
$w_{E,PV}$	8.0 nS
$w^d_{E,SST}$	4.0 nS

Table 2: **Parameters of the network with inhibitory subtypes.**

NMDA conductances

In Suppl. Fig. S7, the excitatory conductance g_E for each neuron i is composed of an AMPA and a an NMDA term as in Zenke *et al.* (2013).

$$g_E(t) = \alpha^{\text{AMPA}} g_i^{\text{AMPA}} + (1 - \alpha_{\text{AMPA}}) g_i^{\text{NMDA}}$$

where

$$\tau^{\text{AMPA}} \frac{dg_i^{\text{AMPA}}}{dt} = -g_i^{\text{AMPA}} + \sum_{j \in E} w_{ij} S_j(t)$$

$$\tau^{\text{NMDA}} \frac{dg_i^{\text{NMDA}}}{dt} = -g_i^{\text{NMDA}} + g_i^{\text{AMPA}}$$

Table 3: **Parameters of the excitatory conductances.**

Parameter	Value
τ^{AMPA}	20 ms
τ^{NMDA}	100 ms
α^{AMPA}	0.5

Synaptic Scaling

In Suppl. Fig. S9, instead of a sliding depression amplitude (Bienenstock *et al.*, 1982), we used synaptic scaling as a homeostatic mechanism, implemented by the term $\gamma(\kappa - \bar{s}_i)$ in the following weight update equations. As before $\bar{s}_i$ is the moving average of neuron i's activity, κ is the target firing rate. For perisomatic synapses, weights change according to:

$$\begin{aligned}\frac{dw_{ij}}{dt} =& \eta w_0 A^+ z_j^+(t) z_i^{slow}(t-\epsilon) S_i(t) \\ &+ (-\eta w_0 A_i^- z_i^-(t) + \gamma(\kappa - \bar{s}_i)) S_j(t)\end{aligned} \tag{4}$$

where

$$\gamma = \frac{A^+ \tau^+ \tau^{\text{slow}}}{\tau^-} \tag{5}$$

Equivalently, for the dendritic synapses, weights change according to:

$$\frac{dw_{ij}^{d}}{dt} = \eta^{d} w_0 A^{+} z_j^{+}(t) z_i^{slow}(t-\epsilon) S_i^{\mathrm{bAP}}(t) + (\eta^{d} w_0(-A_i^{-} z_i^{-}(t) + A^{Ca}(v_d > \theta_{Ca})) - \alpha + \gamma(\kappa - \bar{s}_i)) S_j(t) \quad (6)$$

A_i^{-} was fixed to 2.7e-3.

Memory network

For Suppl. Fig. 10 we used the described network and added plasticity on inhibitory to excitatory synapses (see section "Inhibitory plasticity") and a competition mechanism for postsynaptic weights.

Heterosynaptic depression If the sum of postsynaptic weights in the perisomatic compartment or the dendrite exceeds a maximum $1.5pN_E w_0$ (hard bound), all perisomatic/dendritic synaptic weights are scaled down equally by the average synaptic weight change of the postsynaptic compartment in the current time step, that is the total perisomatic/dendritic weight change divided by the number of incoming perisomatic/dendritic synapses.

Stimulation protocol We first simulated the non-plastic network for 5 s to calculate the steady state population firing rate. We then introduced plasticity with a target firing rate κ of the measured population firing rate and simulated the network with plasticity for another 5 s. Then, we activated pattern P1 for 3 s, which was realised by an external input of 100 Poisson spike trains with a firing rate of 20 Hz to neurons with indices 400 to 499 (ensemble E1). Afterwards, we changed the gating variable under investigation (except in Fig. 10a, where we did not apply any gating). After a stimulation pause of 7 s, we activated pattern P2 for 5 s, which was realised by an external input of 100 Poisson spike trains with a firing rate of 30 Hz to neurons with indices 450 to 549 (ensemble E2). We continued the simulation for further 10 s without stimulation.

Supplementary Figures

Dendritic weight changes as a function of τ_{crit} in the network simulations shown in Fig. 2e

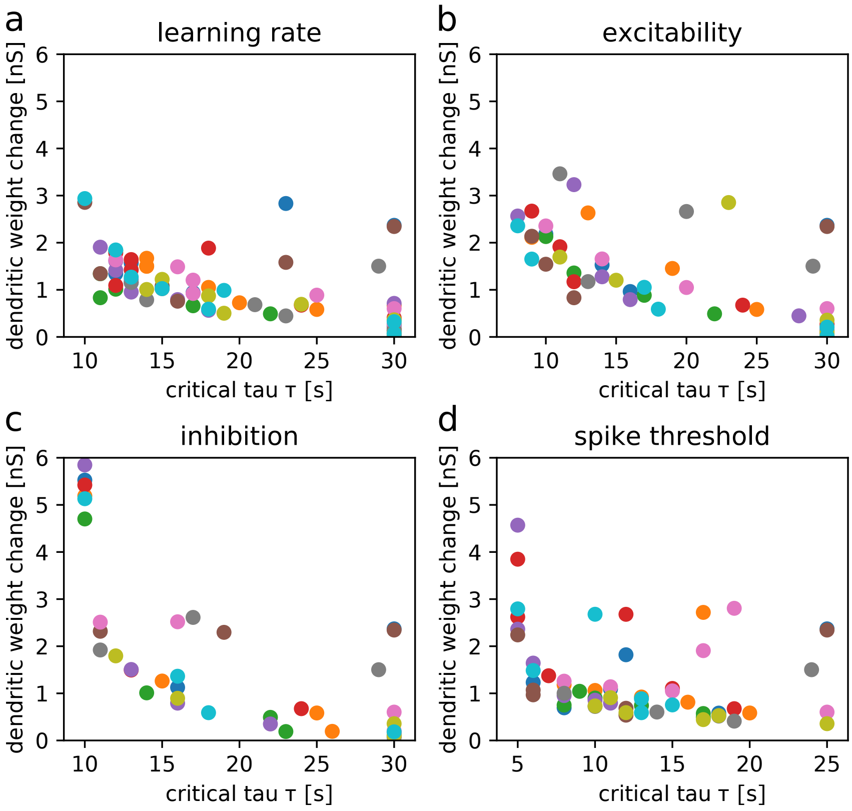

S 1. Dendritic weight changes as a function of τ_{crit} in the network simulations shown in Fig. 2e where learning rate (a), excitability (b), inhibition (c), and spike threshold (d) were varied. The different colours represent the 10 different seeds used for the simulations.

Combinations of different gating variables.

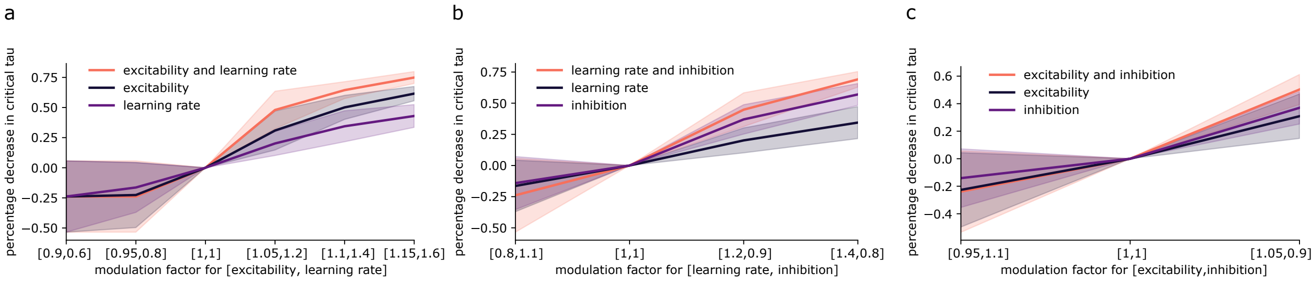

S 2. Combinations of different gating variables. We simulated the network with different values for each gate and combinations of two gates at a time to compare how their effects add up. We measured the decrease of the critical time constant in percent compared to its value in a default network (with modulation factors [1,1]). Shown is by which percentage the critical time constant decreases with a change in each gate or both together. The x-axis denotes by which modulation factor each gate was changed. Learning rate and excitability increase, whereas inhibition decreases from left to right.

Dendrites and inhibitory plasticity

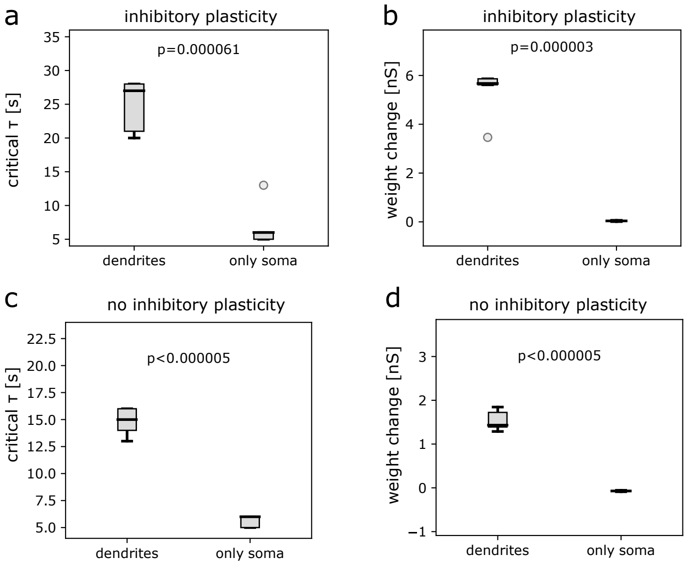

S 3. Dendrites and inhibitory plasticity. Here, we are comparing a model with neurons with dendrites to a model with neurons without dendrites (only soma). To make the two models comparable, we add the dendritic (excitatory and inhibitory) synapses to the soma in the soma only model with the same connection probability and strength as in the model with dendrites. Then we increase the learning rate by a factor of two of those excitatory synapses on the dendrite (in the model with dendrites) and of those excitatory synapses that used to be on the dendrite (in the model without dendrites). Similarly, we add inhibitory plasticity (see Suppl. Methods) to the inhibitory synapses on the dendrites (in the model with dendrites) and to the inhibitory synapses on the soma that used to be on the dendrite (in the model without dendrites). Inhibitory plasticity in the model with dendrites (a) increases both the tolerated critical time constant (compare to c without inhibitory plasticity), and it allows for more synaptic change in the dendrite (b, compare to d). In the model without dendrites however, the stability does not change very much with the same amount of inhibitory plasticity (compare only soma in a and c) and the synaptic weight changes are remain small (compare only soma in b and d).

Effect of the dendritic nonlinearity.

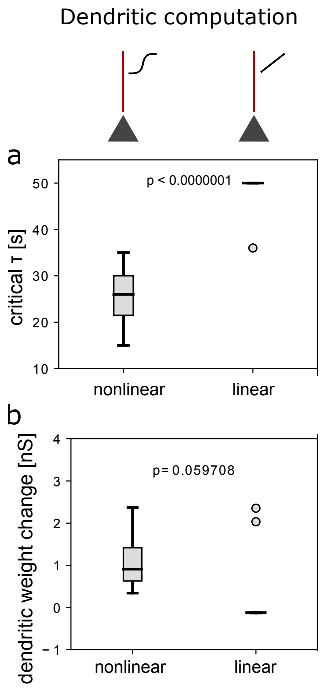

S 4. Effect of the dendritic nonlinearity. a: Distribution of critical homeostatic time constants for a network with nonlinear dendrites and a network with linear dendrites. b: Distribution of dendritic weight changes for a network with nonlinear dendrites and a network with linear dendrites. The rectangles represent the interquartile range (IQR) between first and third quartiles. The thick horizontal lines represent the medians. The whiskers indicate the lowest and highest values within 1.5xIQR from the first and third quartiles, respectively. The circles denote outliers. p-values were obtained by using the two-sample two-sided student's t-test.

Comparison of gating plasticity in the dendrites in a network with two-compartment neurons to gating plasticity in the perisomatic compartment in a network with single-compartment neurons.

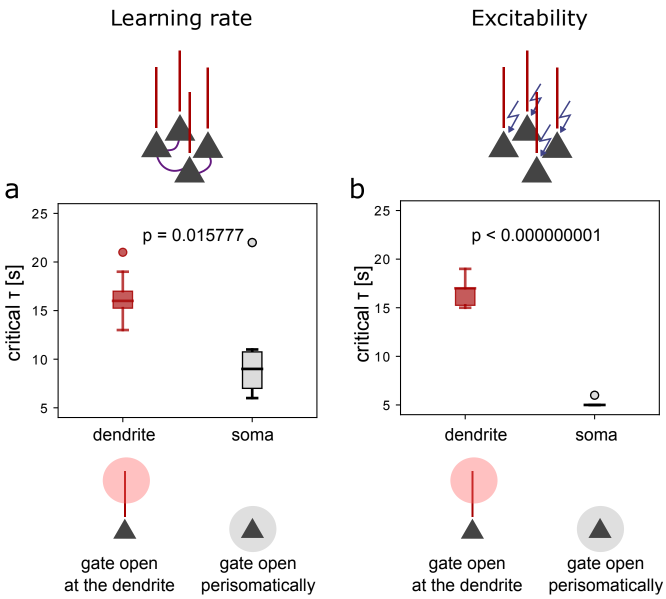

S 5. Comparison of gating plasticity in the dendrites in a network with two-compartment neurons to gating plasticity in the perisomatic compartment in a network with single-compartment neurons. Distribution of critical homeostatic time constants for gating in the dendritic (red) and in the perisomatic (black) synapses for (a) a two-fold increase in the learning rate, and (b) a 15% increase in excitability. The rectangles represent the interquartile range (IQR) between first and third quartiles. The thick horizontal lines represent the medians. The whiskers indicate the lowest and highest values within 1.5xIQR from the first and third quartiles, respectively. The circles denote outliers. All p-values were obtained by using the two-sample student's t-test.

Feedforward and feedback inhibition by different inhibitory subtypes

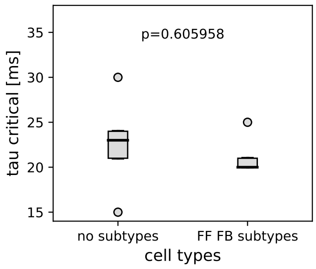

S 6. Feedforward and feedback inhibition by different inhibitory subtypes We simulated a network with two inhibitory cell types, one population targets the dendrites, and another population of inhibitory cells targets the soma. Only the soma-targeting population (presumably parvalbumin-positive cells) receive the feedforward inputs, which are also the feedforward inputs of the excitatory cells. The dendrite-targeting population (presumably somatostatin-positive cells) only receive feedback inhibition from the excitatory cells. We compared this new network with inhibitory subtypes to the original network and do not find a significant difference for the stability.

Role of NMDA channels

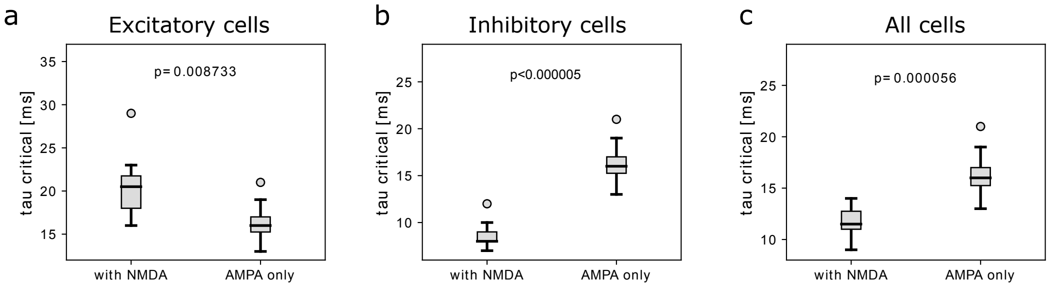

S 7. Role of NMDA channels. To investigate how NMDA receptors change the network stability, we added NMDA conductances to either the excitatory cells, the inhibitory cells, or both. Increasing the NMDA ratio (from 0% to 50%) only in the excitatory cells indeed increases stability (a). However, increasing the NMDA ratio (to 50%) in inhibitory cells (b) or in both excitatory and inhibitory cells (c) decreases stability.

Dependence of the critical homeostatic time constant on parameters.

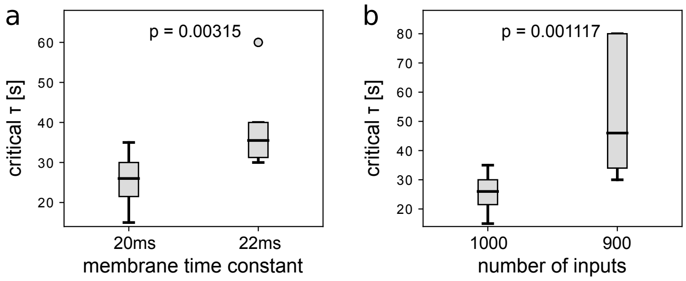

S 8. Dependence of the critical homeostatic time constant on parameters. a: The network was simulated with two different membrane time constants. For each condition, the network was simulated 10 times with different seeds. The distribution of critical homeostatic time constants is plotted for the network with a membrane time constant of 20ms, and for the network with a membrane time constant of 22ms. b: The network was simulated with different numbers of excitatory Poisson inputs. Both the excitatory and the inhibitory cells receive these inputs. The distribution of critical homeostatic time constants is shown for the network with 1000 Poisson inputs, and for the network with 900 Poisson inputs. The rectangles represent the interquartile range (IQR) between first and third quartiles. The thick horizontal lines represent the medians. The whiskers indicate the lowest and highest values within 1.5xIQR from the first and third quartiles, respectively. The circles denote outliers. p-values were obtained with the two-sample student's t-test.

The network with synaptic scaling as a homeostatic mechanism.

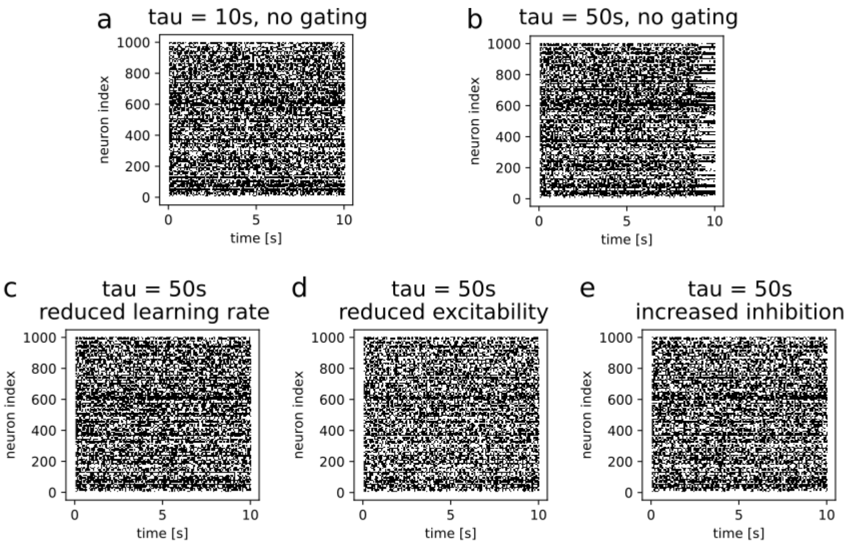

S 9. The network with synaptic scaling as a homeostatic mechanism. Raster plots of a network with synaptic scaling as a homeostatic mechanism. a: no gate applied, synaptic scaling with a homeostatic time constant of 10s. b-e: synaptic scaling with a homeostatic time constant of 50s. b: no gate applied. c: reduced learning rate (reduced by 40%). d: reduced excitability (reduced by 10%). d: increased inhibition (increased by 10%).

Plasticity-inducing stimulation of the network and protection of memories by gating plasticity.

In addition to the impact on the stability of network activity, plasticity interferes with the stability of memories. In a plastic network, neural activity patterns lead to synaptic changes, which could overwrite memories that were previously stored in the synaptic connections. Especially when resources are limited, forming new memories can come at the expense of old ones. By gating plasticity, memories can in principle be protected. For example, trivially, by switching off plasticity, old memories are protected. Interestingly, the gates we considered here are not simple all-or-nothing gates but can be continuously modulated. We, therefore, investigated how their modulation affects the maintenance and encoding of memories. Note that we do not use spatially localised gating here.

Before gating plasticity, we first tested how a memory interferes with a previously stored memory in a plastic network. We added inhibitory plasticity (Vogels *et al.,* 2011) to ensure network stability during memory formation and heterosynaptic depression to introduce synaptic competition (see Suppl. Methods). First, we showed pattern P1 to the network (Fig. S10a). That is, a subpopulation of 100 excitatory neurons received excitatory Poisson input (100 Poisson spike trains with a firing rate of 20 Hz). Afterwards, neurons activated by P1 formed a neural ensemble E1 by increasing their connectivity (Fig. S10a top left). Then, we showed a pattern P2 to the network. P2 is similar to P1 and hence activated a group of neurons E2 that overlapped with the previously formed ensemble. Neurons activated by P2 increased their connectivity. Because the patterns overlapped, synapses were increased at the expense of connections from the old memory (Fig. S10a bottom). Therefore, the new memory interfered with the old memory (Fig. S10 top right). We defined the difference between the mean connection strength of the P1 neurons after memory formation of P1 and the mean connection strength of the P1 neurons at the end of the simulation - after P2 has been learned - as the *breakdown* of the memory.

To test the effect of gating on the protection of memories, we applied the different gates after the first memory is formed. Reducing learning rate to 0 after the first memory is formed trivially protects the memory from being overwritten by the second pattern (Fig. S10b), as this blocked further weight changes. It is less clear how a change in excitability or inhibition affect the storage of the memory. Unlike the learning rate, we cannot modulate excitability or inhibition to their extremes without silencing neural activity. On the one hand, because they decrease neural firing rates, these gates could protect memories by reducing weight changes. On the other hand, by decreasing firing rates, they could also increase LTD as experimentally, low firing rates promote more LTD than LTP (Dudek and Bear, 1992; Sjöström *et al.* 2001).

We found that a reduction in excitability could indeed protect the memory (Fig. S10c) without permanently silencing the network (Fig. S11c). With lower excitability, the stimulated neural ensemble, E2, fired at a lower rate (compare E2 in Figs. S11g and S11e), and weights within E2, including those projecting to the overlapping ensemble, O, potentiated less (compare Fig. S10c bottom with Fig. S10a bottom). The new ensemble, E2, hence competed to a lesser extent with the old memory, leading to less memory decay due to heterosynaptic depression. Note that protecting the old memory hence comes at the cost of storing an equally sized representation of P2.

Similar to reduced excitability, increased inhibition could also protect the memory (Fig. S10c) as it reduced firing rates in the network (Fig. S9d,h). Notably, the inhibitory plasticity in the network additionally protected the previously formed memory, as it led to increased inhibition of the old memory ensemble E1 (Fig. S11i). This further reduced potentiation of synapses from the new ensemble, E2, to the overlapping ensemble, O.

Because decreased excitability and increased inhibition lower the firing rate of the network, we asked whether this low firing rate induces LTD and hence counteracts the protection of the old memory ensemble. We found that there was no increased LTD within the old memory ensemble due to low firing rates. First, the non-overlapping population of the old ensemble fired at a very low rate (E1-O in Fig. S11g,h). There was hence little depression from the non-overlapping population to the overlapping one, as depression happens upon presynaptic spiking. Second, the memory breakdown was weaker at lower excitability and higher inhibition, i.e. at lower firing rates (Fig. S10f,g). The old memory was hence mostly at risk due to heterosynaptic depression. In line with this, the memory breakdown correlated with the maximum mean strength of synaptic connections to the overlapping ensemble O during pattern P2 (Fig. S11j-l).

The specificity of the protective effect depends on which gating mechanism is used. For the learning rate, the effect is specific at the synaptic level. For excitability and inhibition, the effect is specific at the cell level. Excitability, inhibition, and learning rate can be modulated at the compartmental level and introduce further specificity there.

When we continuously modulated the gates, we found that the breakdown of the memory increased nonlinearly with both increasing excitability (Fig. S10f) and decreasing inhibition (Fig. S10g), and increased linearly with learning rate (Fig. S10e).

To conclude, all gates can protect memories. Learning rate can be modulated independent of network activity and hence act as a switch for plasticity. Although excitability and inhibition do not modulate plasticity separately, they can both protect memories by reducing activity and weight changes without silencing network activity.

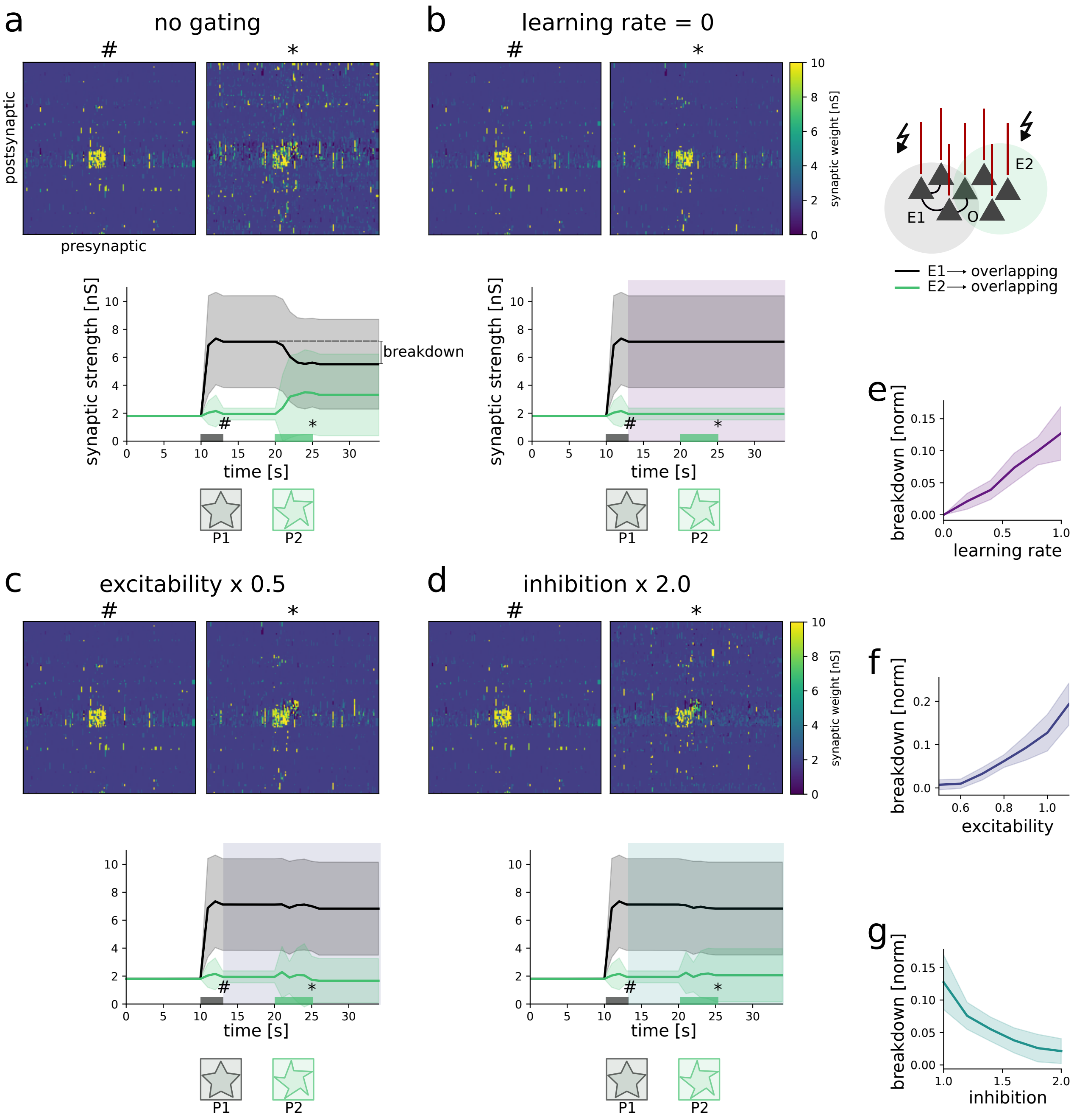

S 10. Plasticity-inducing stimulation of the network and protection of memories by gating plasticity. a-d: After 10 seconds, we show pattern 1 (P1, grey) to the network for 3 seconds. After a gap of 7 seconds, we show pattern 2 (P2, green) to the network for 5 seconds. Top panels: excitatory weight matrix at two time points of the simulation. Bottom panel: mean synaptic weight from ensemble 1 (E1) to the overlapping region (grey) and from ensemble 2 (E2) to the overlapping region over time as an indication of the strength of the memory of P1 and P2, respectively. a: no gating. b: after P1 is learned, the learning rate is set to 0 (denoted by purple background). c: after P1 is learned, excitability is reduced by 50% (denoted by blue background). d: after P1 is learned, inhibition is doubled (denoted by green background). e-g: breakdown of the memory (see a) as a function of learning rate (e), excitability (f), and inhibition (g).

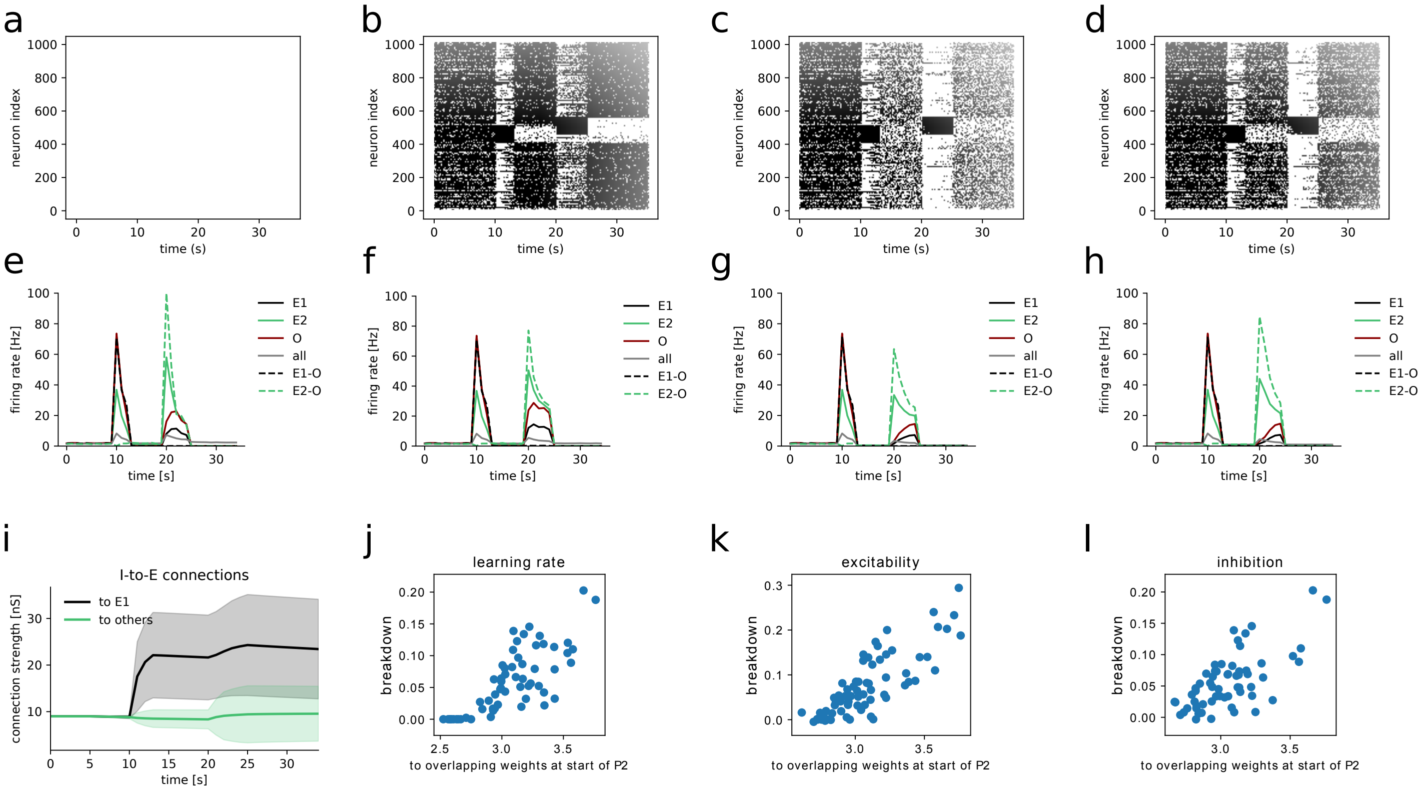

S 11. a-d: Raster plots for the simulation without gating (a), with gated learning rate (b), with gated excitability (c) and with gated inhibition (d) as in Fig. 5e-h: Firing rates over time of different subpopulations: E1: memory ensemble E1 ($x \in E1$), E2: memory ensemble E2 ($x \in E1$), O: overlapping ensemble O ($x \in (E1 \cap E2)$, all: all excitatory neurons in the network, E1-O: ensemble E1 excluding the overlapping ensemble O ($x \in (E1 \setminus O)$), E2-O: ensemble 2 excluding the overlapping ensemble ($x \in (E2 \setminus O)$). i: Connection strengths over time from the inhibitory population to ensemble E1 (black) and from the inhibitory population to all other cells in the ungated network. j-l: Memory breakdown as a function of the maximum during pattern P2 of the mean strength of synaptic connections from all excitatory cells to the overlapping ensemble O for a network with gated learning rate (j), gated excitability (k) and gated inhibition (l).

References

1. E. Bienenstock, L. Cooper, P. Munro, *Journal of Neuroscience* **2**, 32–48, ISSN: 0270-6474, eprint: `http://www.jneurosci.org/content/2/1/32.full.pdf`, (`http://www.jneurosci.org/content/2/1/32`) (1982).

2. T. P. Vogels, H. Sprekeler, F. Zenke, C. Clopath, W. Gerstner, *Science* **334**, 1569–1573, ISSN: 0036-8075, eprint: `https://science.sciencemag.org/content/334/6062/1569.full.pdf`, (`https://science.sciencemag.org/content/334/6062/1569`) (2011).

3. T. Vogels *et al.*, *Frontiers in Neural Circuits* **7**, 119, ISSN: 1662-5110, (`https://www.frontiersin.org/article/10.3389/fncir.2013.00119`) (2013).

4. F. Zenke, G. Hennequin, W. Gerstner, *PLOS Computational Biology* **9**, 1–14, (`https://doi.org/10.1371/journal.pcbi.1003330`) (Nov. 2013).
